# Protein kinases associated with the yeast phosphoproteome

**DOI:** 10.1186/1471-2105-7-47

**Published:** 2006-01-31

**Authors:** Ross I Brinkworth, Alan L Munn, Boštjan Kobe

**Affiliations:** 1 School of Molecular and Microbial Sciences, University of Queensland, Brisbane 4072, Australia; 2 Institute for Molecular Bioscience and Special Research Centre for Functional and Applied Genomics, University of Queensland, Brisbane 4072, Australia; 3 School of Biomedical Sciences, University of Queensland, Brisbane 4072, Australia

## Abstract

**Background:**

Protein phosphorylation is an extremely important mechanism of cellular regulation. A large-scale study of phosphoproteins in a whole-cell lysate of *Saccharomyces cerevisiae *has previously identified 383 phosphorylation sites in 216 peptide sequences. However, the protein kinases responsible for the phosphorylation of the identified proteins have not previously been assigned.

**Results:**

We used Predikin in combination with other bioinformatic tools, to predict which of 116 unique protein kinases in yeast phosphorylates each experimentally determined site in the phosphoproteome. The prediction was based on the match between the phosphorylated 7-residue sequence and the predicted substrate specificity of each kinase, with the highest weight applied to the residues or positions that contribute most to the substrate specificity. We estimated the reliability of the predictions by performing a parallel prediction on phosphopeptides for which the kinase has been experimentally determined.

**Conclusion:**

The results reveal that the functions of the protein kinases and their predicted phosphoprotein substrates are often correlated, for example in endocytosis, cytokinesis, transcription, replication, carbohydrate metabolism and stress response. The predictions link phosphoproteins of unknown function with protein kinases with known functions and vice versa, suggesting functions for the uncharacterized proteins. The study indicates that the phosphoproteins and the associated protein kinases represented in our dataset have housekeeping cellular roles; certain kinases are not represented because they may only be activated during specific cellular responses. Our results demonstrate the utility of our previously reported protein kinase substrate prediction approach (Predikin) as a tool for establishing links between kinases and phosphoproteins that can subsequently be tested experimentally.

## Background

*Saccharomyces cerevisiae *expresses over 110 Ser/Thr protein kinases that have been classified into 7 groups [1] (Table 1, Additional file 1). Most human protein kinases have orthologues in yeast, including protein kinase A (PKA), protein kinase C (PKC), Akt (PKB), calcium/calmodulin-dependent kinase type II (CaMK2), 5'-AMP activated kinase (AMPK), cyclin-dependent kinases (CDKs), mitogen-activated protein kinases (MAPKs), glycogen synthase kinase 3β (GSK3β), p21-activated kinase (PAK1), polo-like kinase (PLK1), mixed lineage kinase (MLK1), and casein kinases 1 (CK1) and 2 (CK2). On the other hand, there are no orthologues of the human protein kinase G (PKG), phosphorylase kinase (PHK), titin, myosin light chain kinase (MLCK), tousled-like kinase (TLK), or any of the protein tyrosine kinases. Some dual-function kinases in yeast can phosphorylate tyrosine, usually at the same time as serine/threonine residues (*e.g. *mitogen-activated protein kinase kinase (MAPKK or MEK) and protein kinase Swe1p [2,3]).

**Table 1:** Classification of protein kinases of S. cerevisiae*

Group or family	Group or family name	Example protein kinase	Number of protein kinases	Number of phosphorylation sites predicted
Group I	AGC kinases (PKA, PKG, PKC)		17	36
I_A, I_C, I_F	cAMP-dependent protein kinases	Sch9p	9	
I_B	Protein kinase C (Pkc1p) family	Pkc1p	1	
I_D	Ribosomal S6 kinase family	Kin82p	2	
I_E	Dbf2p family	Dbf2p	2	
I_G	Other AGC kinases	Yfl033cp	3	
Group II	CaMKs		17	24
II_A	Kinases regulated by calcium/calmodulin	Cmk1p	4	
II_B	5'AMP activated kinase family	Snf1p	6	
II_C	Nim1p family	Hsl1p	3	
II_D	Other CaMKs	Dun1p	4	
Group III	Pro-directed (CMGC) kinases		21	23
III_A	Cyclin-dependent kinases	Cdc28p	5	
III_B	Mitogen-activated kinases (MAPKs)	Slt1p	6	
III_C	Glycogen synthase kinase 3β	Mds1p	4	
III_D	CDK-like kinases (CLKs)	Yak1p	4	
III_E	Other CMGC kinases	Sgv1p	2	
Group IV	Ste11p/Ste20p (MAPKKKs)		10	26
IV_A	Ste11p family	Bck1p	4	
IV_B	Ste20p family	Cla4p	3	
IV_C	NRK Family	Cdc15p	3	
Group V	Ste7p/NEK (MAPKKs)		8	32
V_A	Ste7p family	Ste7p	4	
V_B	NIMA (NEK) family	Kin3p	1	
V_C	NEK-like family	Akl1p	3	
Group VI	Other kinases		24	96
VI_A	CK1 family	Yck1p	4	
VI_B	CK2 family	Cka1p	3	
VI_C	Npk1p/Hal5p family	Ptk1p	9	
VI_D	Elm1p family	Pak1p **	3	
VI_E	Ran1p family	Sha3p	3	
VI_F	Pim1p-like family	Yal017wp	2	
Group VII	Unique kinases		19	38
VII_A	With possible homologues	Rad53p	14	
VII_B	With no known homologues	Bub1p	5	

* Based on [1].

** There are two protein kinases designated Pak1p in *S. cerevisiae*; in this manuscript, Pak1p from group VI_D is referred to as Pak1p (Yer129wp), while Pak1p from group V_C is referred to as Prk1p (Pak1p).

A large-scale mass spectrometry-based study of phosphoproteins in a whole-cell lysate of *S. cerevisiae *detected 383 phosphorylation sites in 216 phosphopeptide sequences [4] (Additional file 2), but the protein kinases responsible for the phosphorylation of these sites have previously remained unknown. We have previously developed methodology to predict the substrates of protein kinases (program Predikin [5]). The predictions are based on the nature of residues located in the substrate-binding pocket at specific positions/distances relative to the conserved motifs found in all Ser/Thr protein kinase sequences. The approach allows predictions to be made based only on the amino acid sequence of the catalytic domain of the kinase. Several studies have confirmed Predikin predictions experimentally, for example in G-protein-coupled receptor kinase [6], stress-activated protein kinase 3 [7], protein kinase C [8], homeodomain-interacting kinase [9] and a tobacco osmotic stress-activated protein kinase [10].

Here we used Predikin to predict possible associations between the phosphorylation sites identified by Ficarro *et al. *[4] and one or more of the yeast protein kinase(s). Some of these predictions are thought-provoking and suggest areas to investigate further. The study suggests biological functions for uncharacterized protein kinases and substrates, forecasts new signalling connections between known proteins, and provides a basis to direct future experimental work to verify the links between kinases and substrates.

## Results and discussion

### Reliability and limitations of the predicted kinase-substrate associations

Ficcaro and co-workers attempted to characterise the majority of the phosphoproteins in a whole cell lysate of *S. cerevisiae *[4]. Proteins were digested with trypsin, converted to methyl esters, enriched for phosphopeptides with immobilised metal-affinity chromatography (IMAC), and analyzed by HPLC/ electrospray ionization mass spectrometry. A total of 383 phosphopeptides were found, corresponding to 216 phosphoproteins (Additional file 2). It appears that phosphopeptides with more than one phosphorylated residue are enriched in this collection, therefore it may not be entirely representative of the yeast phosphoproteome. We attempted to associate these phosphorylation sites with 116 Ser/Thr protein kinases present in *S. cerevisiae *(Additional file 1, 3), through matching the phosphopeptide sequences with the substrate specificity of each individual kinase as predicted using Predikin [5].

**Figure 1:**
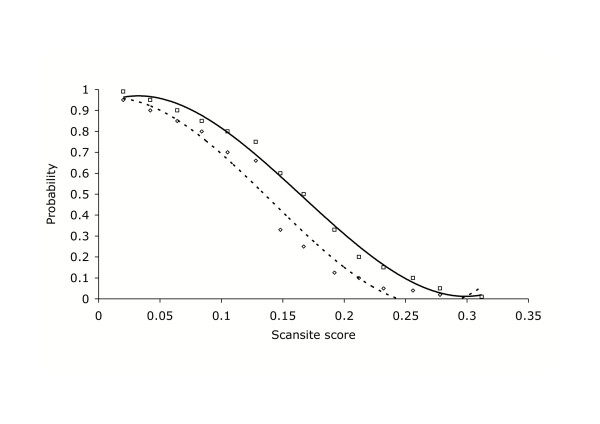
**Estimation of kinase substrate prediction probabilities**. The analysis is based on Scansite [11] scores. Diamonds, the probability (A) that a phosphorylation site is associated with a particular protein kinase, where the protein is a known substrate but the phosphorylation site is unknown, as a function of Scansite score. Squares, the probability (B) that a protein kinase is associated with a particular substrate protein, where the phosphorylation site is known but the protein kinase is unknown, as a function of Scansite score. The lines are 3rd order polynomials that best fit the data (dashed, probability A; solid, probability B).

We used Scansite [11] to assess the quality of the match between the search motif (the predicted optimal heptapeptide phosphorylation sequence for each kinase) and the experimentally determined phosphopeptide sequences. To estimate the reliability of the protein kinase- phosphoprotein matches, we performed an analysis of Scansite scores for phosphorylation sites where the kinase has been experimentally identified (see Methods section; Figure 1).

Scansite scores could be obtained and probabilities estimated for all but 27 of the phosphorylation sites in the phosphoproteome dataset; one cannot obtain an accurate score when the phosphorylation site is located too close to the N- or C-terminus of the peptide. The statistics on estimated probabilities are summarized in Table 2.

**Table 2:** Statistics on the prediction probabilities

Estimated probability	Number of phosphorylation sites
0.9 – 0.99	48
0.80 – 0.89	89
0.70 – 0.79	54
0.60 – 0.69	69
0.50 – 0.59	32
<0.5	55

In some cases, two or more kinases were predicted to be equally likely to be associated with a particular phosphopeptide. Often, these protein kinases were closely related (e.g. from the same family in the phosphopeptides from Hxt2p, Ras2p, Rpl7Ap, Ira2p and Nup2p; from the same group in Acc1p, Gnp1p, Fpr4p, Hsp26p, Pea2p, Rpn8p, Shp1p, Yhr186cp, Yml029wp, Ynl321wp, Ysc84p), although this was not always the case (e.g. phosphopeptides from Abf1p, Bud4p, Erg6p, Msc3p, Ncb2p, Sgv1p, Trs120p, Tsl1p, Yml072cp). Some sites may in fact be phosphorylated by two or more protein kinases *in vivo*, particularly by closely related protein kinases. An example of a substrate phosphorylated by two different kinases is the yeast amphiphysin homologue Rvs167p, which is phosphorylated both by the CDK Pho85p and the MAPK Fus3 [12].

In the cases where both the phosphoprotein and the predicted kinase have characterized functions, correlations between these functions are consistent with and support (although do not necessarily prove) our predictions (see below). On several occasions the phosphoproteins scored better with poorly characterized protein kinases than with any better-known ones; such associations could predict functions for these novel proteins.

### Known protein kinase-substrate pairs

Although our associations were predicted without taking into account the available data on the functions of the proteins, the predictions include several examples of protein kinase-substrate pairs for which there is supporting experimental evidence (Table 3). These include Yak1p [13] and Sra1p [14,15] as substrates for Tpk1p, and Acc1p [16] and Pfk2p [17] as substrates for Snf1p. The MAPKs Kss1p and Fus3p, closely related to Hog1p, have been shown to be substrates for Ste7p [18]. The MAPKK Pbs2p is known to be a substrate for the MAPKKKs Ssk2p and Ssk22p [19], but phosphopeptides corresponding to the known Ssk2p and Ssk22p phosphorylation sites of Pbs2p were not detected by Ficarro *et al. *[4]; the phosphopeptides identified by Ficarro et al. map to a different region of Pbs2p.

**Table 3:** Predicted protein kinase- phosphoprotein pairs supported by experimental evidence

**Substrate protein**	**Predicted protein kinase**	Phosphopeptide *	Scansite score **	Estimated probability ***
Yak1p	Tpk1p	*R*RKSSLVV	0.145, 0.02	0.6, 0.95
Yak1p	Tpk1p	*R*RASLNS	0.127	0.75
Sra1p	Tpk1p	RSRSSVM	0.103	0.8
Acc1p	Snf1p	*R*AVSVSD	0.145	0.6
Pfk2p	Snf1p	*K*VHSYTD	0.145	0.6
Hog1p	Ste7p	PQM**T**GYVST	0.19	0.33
Bni5p	Cdc28p	PVSSPIT	0.145	0.6
Gpd1p	Bck1p	*R*SSSSV**S**LK*A*	0.141	0.6
Hsp26p	Ume5p	LANTPAK	0.082	0.85
Msn2p	Pkc1p	*R*RPSYR*R*	0.147	0.6
Sok2p	Yak1p	*K*SISPR*T*	0.148	0.6
Ssd1p	Hog1p	SLSSPTK	0.039	0.95
Ste2p	Fus3p	QLP**T**PTSSKN	0.04	0.99

* The phosphorylated residues are underlined, and the residues not present in the phosphopeptide sequences [4] are shown in italic. When there is more than one phosphorylation site, the one discussed is shown in bold, unless the same protein kinase is predicted for all sites in the peptide.

** Scansite [11] scores were calculated as described in the Methods section. When the same protein kinase is predicted for all sites in the peptide, the scores are given for the respective sites, starting at the N-terminus. If more than one protein kinase yields a similar score, all the possible kinases are listed.

*** Probabilities were calculated as described in the Methods section (Figure 1). When the same protein kinase is predicted for all sites in the peptide, the values are given for the respective sites, starting at the N-terminus.

Less direct experimental evidence has previously suggested the association of several other predicted kinase-substrate pairs (Table 4). For example, Bni5p is a part of a multi-protein complex involving the protein kinase Gin4p and the septins, and the protein kinase Cdc28p is required for the association of Gin4p with the septins [20]. Overexpression of Gpd1p reduces the hypersensitivity to osmotic shock of a hypersensitive yeast mutant, presumably due to the effect on signalling through the protein kinase C-MAPK pathway involving Bck1p; the over-expression of Bck1p similarly restored the protein kinase C signalling to the same mutant and rescued its hypersensitivity to osmotic shock [21]. The protein kinase Ume5p (Ssn3p, Srb10p) is known to be involved in the transcriptional repression of the *HSP26 *gene [22]. Msn2p is a protein kinase Pkc1p-regulated transcription factor [23]. Inactivation of the protein kinase Yak1p and overexpression of Sok2p have similar effects on yeast defective in protein kinase A signalling [24], suggesting that phosphorylation by Yak1p could have an inhibitory effect on Sok2p. The inactivation of protein kinases Hog1p and Ssd1p have similar effects on mutants deficient in the protein phosphatase activators Rrd1p and Rrd2p [25]. Finally, mutation of the gene encoding the protein kinase Fus3p (a MAPK in the mating pathway) enhances the mating defect of some *ste2 *mutants; the deletion of *STE2 *is completely sterile, therefore these are weak alleles of *ste2 *[26]. The available experimental evidence for all these predicted associations supports the reliability of our predictions in general.

**Table 4:** Selected protein kinase- phosphoprotein pairs showing functional correlations

Substrate protein	Function *	Predicted protein kinase	Function *	Phosphopeptide **	Scansite score ***	Estimated probability ****
Cdc47p	DNA replication	CK2 group *****	G1/S and G2/M transition of mitotic cell cycle, cell ion homeostasis, cell polarity, flocculation, regulation of transcription (Pol I and Pol III promoters), response to DNA damage, regulation of DNA replication	TMDTDQE	0.212	0.25
Ede1p	Endocytosis	CK1 group ******	Cellular morphogenesis, cytokinesis, endocytosis, DNA repair, cell growth, chromosome segregation, meiosis, mitosis, nuclear division	DGE**S**VSSIQ	0.062	0.9
Ysc84p	Actin organization, endocytosis	CK1 group	Cellular morphogenesis, cytokinesis, endocytosis, DNA repair, cell growth, chromosome segregation, meiosis, mitosis, nuclear division	DFDSEDE	0.062	0.9
Pan1p	Endocytosis, cytokinesis, budding	CK1 group	Cellular morphogenesis, cytokinesis, endocytosis, DNA repair, cell growth, chromosome segregation, meiosis, mitosis, nuclear division	ASASSTS	0.1	0.8
Sec4p	Rab GTPase (cytokinesis, exocytosis, polar budding)	CK1 group	Cellular morphogenesis, cytokinesis, endocytosis, DNA repair, cell growth, chromosome segregation, meiosis, mitosis, nuclear division	RTVSAS**S**GNG	0.082	0.85
Sec3p	Cytokinesis, cell polarity	Cdc15p	Cytokinesis, regulation of exit from mitosis	RTISGS**S**AHH	0.232	0.15
Hsp26p	Chaperone, stress response	Mds1p	Proteolysis, response to stress, sporulation	EVS**S**QESWGN	- *******	
Nth1p	Trehalose hydrolysis, stress response	Yfl033cp	Regulation of meiosis, stress response	RRGSEDD	0.082	0.85
Hxt2p	Hexose transport	Sha3p	Glucose transport, transcriptional regulation (Pol II promoter)	QQTSIH**S**TPI	0.275	0.05
Shp1p	Glycogen metabolism, sporulation	Snf1p	AMP-activated protein kinase, cell adhesion, response to nitrogen starvation, filamentous growth, glucose metabolism, regulation of carbohydrate metabolism	RKG**S**TSPEP	0.145	0.6
Tps3p	Trehalose phosphatase (carbohydrate metabolism, stress response)	Snf1p	AMP-activated protein kinase, cell adhesion, response to nitrogen starvation, filamentous growth, glucose metabolism, regulation of carbohydrate metabolism	RTS**S**SMSVGN	0.102	0.8
Mcm3p	DNA helicase, initiation of DNA replication	Sgv1p	Transcription	NSGSPIK	0.082	0.85
Swi4p	Transcription factor (cell cycle)	CK2 group	G1/S and G2/M transition of mitotic cell cycle, cell ion homeostasis, cell polarity, flocculation, regulation of transcription (Pol I and Pol III promoters), response to DNA damage, regulation of DNA replication	KSTSETS	0.168	0.5
Rpa190p	RNA polymerase subunit (transcription)	CK2 group	G1/S and G2/M transition of mitotic cell cycle, cell ion homeostasis, cell polarity, flocculation, regulation of transcription (Pol I and Pol III promoters), response to DNA damage, regulation of DNA replication	DKESDSDSEDE	0.104, 0.06, 0.02	0.8, 0.9, 0.99
Mob1p	Protein kinase regulation (cell cycle regulation)	Ume5p	Cyclin-dependent protein kinase, meiosis, regulation of transcription (Pol II promoter)	VLTTPKR	0	0.99
Abp1p	Actin binding (cell polarity)	Cdc28p	Cyclin-dependent protein kinase, regulation of mitosis and meiosis	PSKSPAP	0.145	0.6
Abp1p	Actin binding (cell polarity)	Cdc28p	Cyclin-dependent protein kinase, regulation of mitosis and meiosis	PVK**T**PSPAP	0.125	0.75
Crn1p	Actin filament organization	Cdc28p	Cyclin-dependent protein kinase, regulation of mitosis and meiosis	APKSP**S**PLK	0.145	0.6
Pea2p	Actin filament organization, cell polarity, polar budding	Hog1p	Actin filament organization, osmoregulation, transcriptional regulation (Pol II promoter)	NTSSPPI	0.102	0.8
Spc98p	Cytoskeleton component (microtubule nucleation, mitotic spindle assembly)	Ssk2p	MAPKKK, actin cytoskeleton organization and biogenesis, osmosensory signaling pathway	ERR**S**MVSSPN	0.121	0.75
Npr1p	Protein kinase (regulation of nitrogen utilization)	Snf1p	AMP-activated protein kinase, cell adhesion, response to nitrogen starvation, filamentous growth, glucose metabolism, regulation of carbohydrate metabolism	RQSSIYS	0.149	0.6
Shp1p	Glycogen metabolism, sporulation	Mds1p	Proteolysis, response to stress, sporulation	RKGST**S**PEP	0.082	0.85
Ydl223cp	Cellular morphogenesis	CK1 group	Cellular morphogenesis, cytokinesis, endocytosis, DNA repair, cell growth, chromosome segregation, meiosis, mitosis, nuclear division	STHSAEH	0.1	0.8
Ydl223cp	Cellular morphogenesis	Hsl1p	Cell cycle regulation (G2/M transition of mitotic cell cycle), cell morphogenesis checkpoint, septin checkpoint)	AEHTPRH	0.127	0.75
Fpr4p	Peptidyl-prolyl cis-trans isomerase	CK1 group	Cellular morphogenesis, cytokinesis, endocytosis, DNA repair, cell growth, chromosome segregation, meiosis, mitosis, nuclear division	EDESESEQE	0, 0.082	0.99, 0.85
Fpr4p	Peptidyl -prolyl cis-trans isomerase	CK2 group	G1/S and G2/M transition of mitotic cell cycle, cell ion homeostasis, cell polarity, flocculation, regulation of transcription (Pol I and Pol III promoters), response to DNA damage, regulation of DNA replication	EDESESEQE	0.084, 0.149	0.85, 0.6

* Functional annotations based on RefSeq [68].

** The phosphorylated residues are underlined, and the residues not present in the phosphopeptide sequences [4] are shown in italic. When there is more than one phosphorylation site, the one discussed is shown in bold, unless the same protein kinase is predicted for all sites in the peptide.

*** Scansite [11] scores were calculated as described in the Methods Section. When the same protein kinase is predicted for more than one site in the peptide, the scores are given for the respective sites, starting at the N-terminus. If more than one protein kinase yields a similar score, all the possible kinases are listed.

**** Probabilities were calculated as described in the Methods section (Figure 1). When the same protein kinase is predicted for more than one site in the peptide, the values are given for the respective sites, starting at the N-terminus.

***** Protein kinases Cka1p, Cka2p or Cdc7p. The predicted specificties are too similar to be distinguished.

****** Protein kinases Yck1p, Yck2p, Yck3p or Hrr25p. The predicted specificities are identical.

******* Scansite score could not be measured because the phosphorylation site is too close to the C-terminus.

### Predicted kinase-substrate pairs showing functional correlations

In addition to the known kinase-substrate pairs, our predicted associations feature a number of pairs consistent with the known roles for the kinase and the substrate (i.e. functional correlations). Indeed, none of our predicted associations involve proteins with functions that are clearly incompatible. The functions of some associated proteins do not show obvious relationships, but instead suggest cross-connections between different cellular pathways. For example, seemingly unrelated processes such as mitosis in growing cells or sporulation/meiosis in starved non-growing cells involve common processes such as chromosome segregation and new cell wall deposition. Our associations provide a number of proposed functional links that can now be experimentally tested. The following examples are representative of the correlated functions in substrate-kinase pairs in the dataset (Table 4).

**Figure 2:**
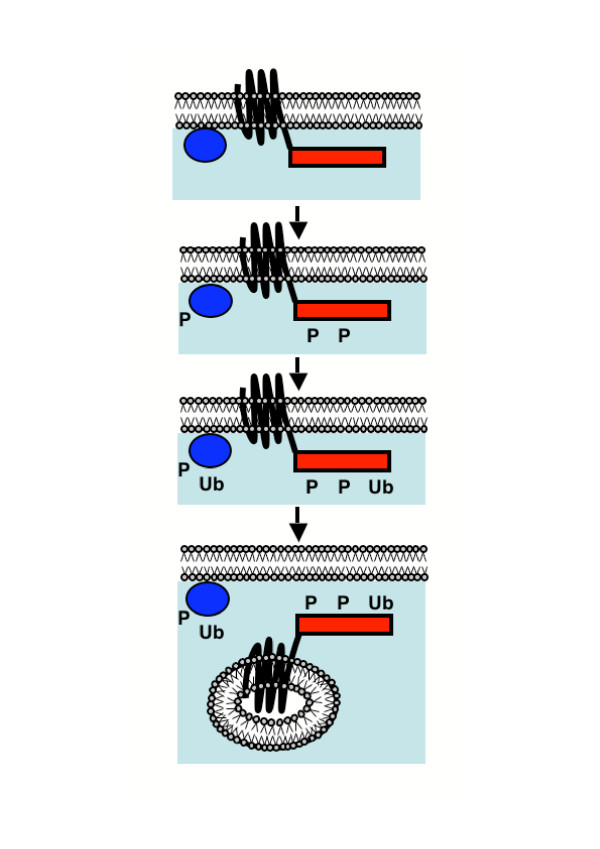
**Model for regulation of endocytosis by phosphorylation and ubiquitination**. In stage 1 (top), neither the receptor (transmembrane region, black; cytoplasmic tail, red rectangle) nor the endocytic machinery (blue circle) are phosphorylated or ubiquitinated. CK1-dependent phosphorylation (P) of the receptor and the endocytic machinery (stage 2, below) leads to Rsp5p-dependent ubiquitination (Ub) of the receptor and the endocytic machinery (stage 3, below), resulting in endocytic internalization of the receptor (stage 4, bottom). Yck1/2p are known to phosphorylate the alpha factor pheromone receptor Ste2p (a 7-transmembrane domain G-protein-coupled receptor) on its cytoplasmic tail [27]. This phosphorylation is essential for ubiquitination of the receptor on its cytoplasmic tail by the ubiquitin protein ligase Rsp5p, and for endocytic internalization of the receptor. There is evidence Rsp5p also has to ubiquitinate components of the endocytic machinery for the receptors to be endocytosed [28]; our analysis of phosphorylation sites suggests that phosphorylation by Yck1/2p of the components of the endocytic machinery (Ede1p, Ysc84p, Pan1p) may play a role also in their Rsp5p-dependent ubiquitination.

Protein kinase CK1 with roles in endocytosis and cytokinesis is predicted to be associated with a number of proteins involved in endocytosis, including Ede1p, a key endocytic protein that binds membranes in a ubiquitin-dependent manner and is involved in a network of interactions with endocytic proteins, the SH3 domain-containing protein Ysc84p, and the actin cytoskeletal protein Pan1p involved in actin cortical actin patch formation. CK1 phosphorylation of receptor cytoplasmic tails [27] is required for subsequent ubiquitination of flanking lysines by Rsp5p [27] and receptor endocytosis. Ubiquitination by Rsp5p of endocytic machinery appears to be required too, as fusion of the receptor cytoplasmic tail to ubiquitin bypasses the requirement for ubiquitination of the receptor cytoplasmic tail, but does not bypass the requirement for Rsp5p in endocytosis per se [28]. Also, Rsp5p physically interacts with Sla1p [29], which in turn interacts with Ysc84p [30] and Pan1p [31]. Furthermore, *RSP5 *genetically interacts with *EDE1 *[32]. Therefore, there is a possibility that CK1 phosphorylation of Ede1p, Ysc84p, and Pan1p is required for their subsequent Rsp5p-dependent ubiquitination on flanking lysines, analogous to what has been shown for receptor cytoplasmic tails. It is not known yet if these proteins are ubiquitinated by Rsp5p; Rvs167p is a component of the endocytic machinery that is known to be ubiquitinated by Rsp5p [29], but it has not been detected among the phosphopeptides. Rvs167p is in a complex with Sla1p and Rsp5p [29] so other Sla1p interactors such as Ysc84p and Pan1p are strong possibilities for Rsp5p-dependent ubiquitination; moreover, the mammalian Pan1p orthologue Eps15 is known to be ubiquitinated [33,34]. A model for the regulation of endocytosis by CK1-dependent phosphorylation and ubiquitination is shown in Figure 2.

CK1 is also predicted to phosphorylate the Rab GTPase Sec4p essential for exocytosis [35]. One yeast CK1 (Yck3p) has been shown to regulate Rab-GTPase dependent vacuole fusion [36,37]. Some endocytic cytoskeletal proteins (e.g. actin, Rvs167p and Sla2p also have roles in the same step of exocytosis as Sec4p [38-40].

Another interesting association involves the major cell cycle regulatory kinase Cdc28p and two actin cytoskeletal proteins, Crn1p and Abp1p. The subcellular distribution of the actin cytoskeleton is tightly regulated during the cell cycle. In late G1 phase and prior to visible formation of a bud, the cells pass "Start"; the Cdc28p kinase becomes activated by G1 cyclins, and the actin cytoskeleton starts to polarise towards the site where the new bud will emerge [41-43]. Cortical actin patches, which appear as highly motile spots, concentrate at this site. Cytoplasmic actin cables, which appear as elongated fibres, exhibit alignment along the mother-bud axis such that their tips are also focused at this site. During S phase, Cdc28p starts to be activated by S-phase cyclins; at this stage of the cell cycle the bud emerges and cortical actin patches concentrate inside the growing bud, especially at the rapidly growing tip. Actin cables remain aligned with their tips in the growing bud, causing the bud to extend in a highly polarised manner. When the cells enter G2 phase, the cortical actin patches distribute more isotropically within the bud, and the bud becomes more rounded and expands both laterally and at the tip. Driven by the mitotic cyclins, maximum Cdc28p activity is achieved and the cells enter mitosis (M-phase). At this stage of the cell cycle cortical actin patches transiently redistribute throughout the mother cell and bud, thus losing their polarisation. The cytoplasmic actin cables become randomly oriented in the mother cell and bud during M phase. Finally, upon exit from mitosis and reduction of Cdc28p activity, the cortical actin patches in the mother cell and the bud align on either side of the bud neck. Cytoplasmic actin cables align with their tips on either side of the bud neck. After cell division is complete and the cells enter early G1, the cortical actin patches again adopt a random distribution and the cytoplasmic actin cables become randomly oriented.

Abp1p is an actin-binding protein that specifically localises to cortical actin patches [44]. Crn1p also localises to cortical actin patches and is thought to act as a linker between these patches and microtubules [45]. Both Abp1p and Crn1p bind to the Arp2/3 complex, a multi-subunit complex that mediates the nucleation step of actin filament assembly within the cortical actin patch. Cortical actin patches are highly motile and short-lived structures. They form by *de novo *actin filament assembly at polarised sites on the cortex where Arp2/3 activators concentrate. Hence their polarisation during the cell cycle is thought to reflect polarisation of the sites where they form. Once assembled, cortical actin patches move rapidly away from these cortical sites and into the body of the cell. This rapid movement is thought to be propelled by the force generated by *de novo *actin filament assembly. One of the Arp2/3 activators implicated in cortical actin patch assembly at the cortex and actin-dependent movement is Abp1p [46]. Abp1p binds to and activates the Arp2/3 complex and thus stimulates *de novo *assembly of actin filaments. Crn1p also binds the Arp2/3 complex. However, in contrast to Abp1p, Crn1p binding to the Arp2/3 complex inhibits (or at least restricts) Arp2/3 activity, in part by preventing Arp2/3 activation by Abp1p [45]. Hence, both Abp1p and Crn1p regulate cortical actin patch formation and dynamics. The association of Cdc28p with Abp1p and Crn1p predicted in this study suggests an important role for these two cortical actin patch components in the response of the cortical actin patches to intrinsic cues generated by Cdc28p, the cell cycle regulatory kinase.

A large proportion of yeast proteins are implicated directly or indirectly in functions such as carbohydrate metabolism, stress, and cell growth. The pairs glycogen synthase kinase Mds1p (Rim11p) – chaperone Hsp26p as substrate, and the protein kinase Yfl033cp (Rim15p) – trehalase Nth1p as substrate have common roles in stress response. The protein kinase Sha3p (Sks1p) and the predicted substrate, the high affinity glucose transporter Hxt2p, share roles in hexose transport, while the AMPK Snf1p and the predicted substrates, the putative regulator of protein phosphatase-1 Shp1p, and the regulatory subunit of trehalose-6-phosphate/synthase/phosphatase complex Tps3p, are all involved in carbohydrate metabolism. Other novel kinase-substrate pairs are also predicted in processes including autophagy, DNA replication and transcription, mitosis and cell cycle, the cytoskeleton, nitrogen utilization, sporulation and cellular growth and morphogenesis (Table 4).

There are several instances of phosphopeptides from protein kinases in the dataset that may indicate protein kinase cascades. MAPKs are indeed identified as MAPKK (group V) substrates (Hog1p and Slt2p predicted as substrates for Ste7p). The other examples include protein kinases Cla4p (predicted as a substrate for the MAPKK Pbs2p and the cell cycle checkpoint kinase Dun1p), Ctk1p (predicted as a substrate for the MAPKK Mkk1p), Npr1p (predicted as a substrate for the AMPK Snf1p; although the two proteins affect different pathways, namely nitrogen and glucose metabolism, respectively), Pbs2p (predicted as a substrate for the MAPKK Mkk1p; although Pbs2p is connected to osmotic sensing but Mkk1p is related to cell integrity sensing), Sgv1p (predicted as a substrate for the cAMP-dependent protein kinase Tpk1p and the uncharacterised protein kinase Yjl057cp), Spc98p (predicted as a substrate for the MAPKKK Ssk2p and MAPKK Pbs2p; Spc98p is a component of the spindle pole body and Pbs2p is connected with osmotic sensing), Yak1p (predicted as a substrate for the cAMP-dependent protein kinase Tpk1p), and the uncharacterized Ydr466wp (predicted as a substrate for the meiosis-specific checkpoint kinase Mek1p and the CDK Cdc28p).

### Prediction of function for novel phosphoproteins and kinases

Several associations are predicted between a protein with a known function and a protein with an unknown function. Putative functional annotations of uncharacterised proteins through such associations represent an intriguing result of this study.

The associations suggest a functional role for a number of uncharacterised protein kinases (Additional file 4). The kinase Akl1p, while uncharacterised, is in the same family as Ark1p and Prk1p, both of which regulate the actin cytoskeleton, and may function in transcriptional regulation, the cell cycle and cell growth-related processes. The kinase Ksp1p, while uncharacterised, is highly homologous to MARK1 (microtubule affinity regulating kinase) in mammals, predicted to function in microtubule cytoskeleton, stress response and polyamine transport. The predictions implicate Kin4p in pH regulation, Kns1p in mating, Ksp1p in phospholipid metabolism, Ygl179cp in pyruvate metabolism, Yjl057cp in transcription, Ypk2p in amino acid transport, meiosis and cell growth, and Ypr106wp in stress response and tRNA processing.

Conversely, the predicted kinases suggest functional roles for a number of phosphoproteins (Additional file 4). For example, protein kinases Pbs2p, Prk1p and Ssk2p jointly implicate the common putative substrate Yhr186cp (Kog1p) in actin organization. Yhr186cp is a component of the TORC complex involving Tor1p or Tor2p; Tor2p is a known regulator of the actin cytoskeleton [47]. Protein kinases Rck1p and CK1 jointly implicate Yhr097cp in meiosis, while the kinases Cdc28p and Mps1p jointly suggest a role in mitosis for Yml072cp. Bre5p, Mlf3p, Yfr016cp and Yro2p may have a role in the cell cycle, Mrh1p in transcription and regulation of meiosis and mitosis, Ydl189wp in meiosis, Ydr090cp in actin and cell wall organization, Ynl136wp in actin filament organization and stress response, Ymr196wp and Yor175cp in stress response, Ycr023cp, Yfr017cp, Yhr132wp, Yml029wp, Ymr295cp, Yor042wp and Yor052cp in cell growth processes, Yfr024cp in amino acid biosynthesis, Ynl156cp and Ynl321wp in cytokinesis, and Ypl247cp in endocytosis.

### Phosphorylation of several sites on the same protein by the same kinase

The predicted association between the kinase-substrate pair may be stronger in the cases where the same kinase is predicted to phosphorylate two or more distinct sites on the same protein (Table 5). Examples of such a kinase-substrate pair are CK1-Ede1p (discussed earlier) and CK1-Tat1p. CK1 is implicated in endocytosis and Tat2p (Tat1p and Tat2p are two highly homologous tryptophan permeases) levels are regulated in response to starvation by endocytosis [48].

**Table 5:** Phosphoprotein-putative protein kinase pairs with several phosphorylation sites attributed to the same kinase

Protein kinase	Substrate protein
Yak1p	Abp1p
Cdc28p	Abp1p
CK2 group*	Dbp10p
Gcn2p	Ede1p
Mck1p	Erg6p
CK1 group**	Ist2p
Mkk1p	Mcm3p
Ume5p	Mlf3p
Yak1p	Msl5p
CK2 group	Nip1p
Hal5p	Not3p
CK1 group	Rpn8p
CK1 group	Sec31p
CK2 group	Sui2p
CK1 group	Tat1p
Tpk1p	Yak1p
CK1 group	Ybt1p
Ume5p	Ydl113cp
CK2 group	Ydl166cp
CK1 group	Ydl223cp
Pbs2p	Ydr384cp
Gcn2p	Yfr024cp
Cmk2p	Ynl156cp
CK2 group	Yro2p

* Protein kinases Yck1p, Yck2p, Yck3p or Hrr25p. The predicted specificities are identical.

** Protein kinases Cka1p, Cka2p or Cdc7p. The predicted specificities are too similar to be distinguished.

### Autophosphorylation

While autophosphorylation may be common in yeast cells, there are only a few protein kinases represented in the phosphoproteome dataset (Akl1p, Cla4p, Hog1p, Ksp1p, Npr1p, Pbs2p, Sgv1p, Slt2p, Yak1p and Ybr466wp), incorporating 17 phosphorylation sites. For 14 of these sites, the specificity of the kinase is quite different from the phosphorylation site, suggesting autophosphorylation is unlikely. Autophosphorylation is most likely in the case of Akl1p, a protein kinase of unknown function from family V_C. This prediction is consistent with yeast two-hybrid analysis [49]. The phosphorylation site (Ser521) is C-terminal to the protein kinase domain (residues 50–400) and is distinct from the activation loop threonine (Thr220), therefore unlikely to involve an auto-activation event. In two other cases involving Cla4p and Pbs2p, autophosphorylation is a possibility, although the phosphorylation sites better match specificities of other yeast kinases. It should be kept in mind that the sequences of autophosphorylation sites can deviate substantially from the usual specificity of the kinase, as a result of the effect of high local concentration during an autophosphorylation reaction [50].

### Tyrosine phosphorylation

Although fungi have no protein kinases classified as protein tyrosine kinases, phospho-tyrosine residues are found in yeast [2,51]. An early example is the protein kinase Spk1p (Rad53p), which can phosphorylate proteins on Ser, Thr and Tyr residues, and can phosphorylate poly(Tyr-Glu) [2,51]. In fact, protein chip experiments showed that 27 yeast kinases can phosphorylate poly(Tyr-Glu) [3], although the physiological relevance of tyrosine phosphorylation by most of these kinases is not yet clear. Currently it is accepted that Tyr phosphorylation in yeast is due to dual specificity protein kinases that can phosphorylate tyrosine in tandem with a nearby threonine [52,53]; they cannot phosphorylate solitary Tyr residues. There are two classes of dual specificity protein kinases in yeast. The first are the MAPKKs (such as Ste7p or Pbs2p) that phosphorylate TXY motifs in the activation loop of MAPKs (in yeast, the MAPKs include Hog1p and Slt2p). The phosphorylation events are carried out in order, with the Tyr phosphorylation occurring first, the Thr phosphorylation occurring second [54]. In our dataset, the topoisomerase-associated protein, Pat1p, has both Ser and Tyr phosphorylated in a SXY motif, which may be the result of MAPKK phosphorylation, or the S6K-like protein kinase Ynr047wp (family I_D), followed by MAPKK phosphorylation. Another example of a dual specificity kinase is Swe1p that phosphorylates both residues at TY sites in CDKs. Cdc28p (the yeast orthologue of CDK2), is a known substrate for Swe1p (the *S. cerevisiae *Wee1 orthologue) [55,56]. No occurrences of dually-phosphorylated TY motifs are present in the phosphoproteome dataset we used.

### Correlations with comprehensive protein association studies in yeast

Several groups attempted comprehensive analyses of protein-protein associations in *S. cerevisiae *(summarized in the Biomolecular Interaction Network Database BIND [57]). A mass spectrometry-based study [58] included 49 yeast protein kinases and 9 proteins from the phosphoproteome dataset as bait proteins. Associations were demonstrated (bait protein listed first) between Hrr25p and Ede1p, and between Rad53p and Ede1p (however, neither phosphorylation site from Ede1p has a good match with Hrr25p or Rad53p specificity); between Ksp1p and Yhr186cp (however, the phosphorylation site matches the specificity of Prk1p to a much greater extent); between Kss1p and Ste11p, between Bck1p and Hog1p (the latter two are MAPK-MAPKKK associations, suggesting they interact through binding an anchoring protein, and do not involve an enzyme-substrate relationship), and between Prk1p with Akl1p (which are closely related and form a family of kinases implicated in actin cytoskeleton regulation). Another mass spectrometry-based study [59] revealed the associations between Chd1p and CK2-type protein kinases Cka1p and Cka2p (consistent with our prediction), between Pkc1p and Eno1p (although Eno1p does not contain a Pkc1p-specific motif), and between Sec31p and both Cka1p and Cka2p (again, neither Sec31p phosphopeptide from the phosphoproteome matches CK2 specificity).

It is not surprising that many protein kinase – substrate pairs cannot be detected as protein-protein complexes, because of the temporary nature of the interaction. The protein association studies using yeast two hybrid methodology [49,60,61] would be expected to reveal more short-term associations than affinity capture-based approaches. A study by Ito and co-workers [60] included a set of 10 *S. cerevisiae *protein kinases as bait, 6 protein kinases as prey, and one kinase as both. Only one pair of associated proteins involved a protein kinase and a protein from the phosphoproteome, protein kinase Ypr106wp (Isr1p) and the protein Chs2p (the catalytic subunit of chitin synthase 2); the association is consistent with our prediction. Only one association with a kinase has been identified in the study by Uetz et al. [49] (protein kinase Chk1p as the kinase with the glycogen synthase Gsy1p).

In summary, few predicted kinase-substrate pairs are supported by comprehensive protein-protein interaction studies. The likely reasons include poor representation of protein kinases and proteins in the phosphoproteome dataset in these studies, and the temporary nature of the kinase-substrate interaction that may be difficult to detect by the methods used in these studies.

Very recently, proteome chip technology has been used to identify the *in vitro *substrates for 82 unique yeast protein kinases, using yeast proteome microarrays containing ~4,400 proteins [62]. This study identified candidate kinases for 50 proteins from the phosphoproteome dataset we used here [4]. Significantly, this study confirmed in 12 cases that our predicted kinases phosphorylate the substrates in vitro, and additionally in 12 more cases closely related kinases were shown to phosphorylate the substrates.

### Representation of different kinase groups among the set of kinases responsible for phosphorylation in the phosphoproteome dataset

The phosphopeptide sequences in the dataset suggest that all 7 major groups of kinases are represented among the kinases responsible for their phosphorylation (Table 1). However, not all the individual protein kinases are represented in our predictions (76 out of 116 were associated with a substrate). There are a number of protein kinases that have apparently phosphorylated a number of the proteins in the dataset, while others are absent altogether. The most frequent kinases to be predicted to phosphorylate sites in the phosphoproteome are the yeast orthologues of mammalian PKA, CaMK2, AMPK, CK1, CK2, and members of the CDK, MAPK and CLK families. Along with PKC, these are the protein kinases that are known to perform housekeeping functions within the cell. We may have been unable to predict all Pkc1p-substrate associations because some Pkc1p-phosphorylated sequences may differ considerably from the optimal Pkc1p phosphorylation consensus motif, and may also fit phosphorylation consensus motifs for other protein kinases (particularly those from group I).

Many protein kinases are linked to specific cellular responses and may only be induced under specific circumstances, and are therefore not necessarily represented in our dataset. Examples of such cellular responses include double-stranded DNA break repair, starvation and mating. The yeast cells used in the study by Ficarro et al. were from a normal growing culture [4] (S. Ficarro, personal communication).

A survey of the functions of the proteins represented in the yeast phosphoproteome shows components of fundamental cellular structures and machines (ribosomes, vacuoles, actin filaments, nuclear pores), intermediary metabolism, endocytosis, cytokinesis, transport proteins, permeases and transcription factors. It is reasonable to imagine that the represented proteins that are as yet uncharacterized will function in these processes; a similar argument can be made for the associated protein kinases that have unknown functions. These kinases often have specificities similar to the better-studied kinases. Our predicted associations are generally consistent with these conclusions.

### Implications for human protein kinases

Few of the proteins in the yeast phosphoproteome dataset have sequences containing the phosphorylation sites conserved in human proteins; many of these proteins do have human homologues, but the phosphorylation site, which is often located near the N- or C-terminus of the protein, has diverged. This observation suggests that the exact location of the phosphorylation site may often be unimportant as a determinant of the regulatory pathway, and that the intricacies of the regulatory mechanisms may differ among species. The following examples illustrate the different cases of conservation between the yeast and human proteins.

In some cases, the sequences surrounding the phosphorylation sites are well conserved, suggesting an equivalent kinase may be responsible for the phosphorylation events in both organisms. Examples include the α-subunit of pyruvate dehydrogenase (phosphorylation site Ser313 in yeast), and glycogen synthase (phosphorylation sites Ser650 and Ser654 in yeast). Neither of the human proteins is known to be phosphorylated at these positions. The yeast MAPK Hog1p and its human orthologue both require double phosphorylation by a MAPKK at a TGY motif [63]. The similarity of the two sequences suggests an equivalent MAPKK is likely to be responsible in both organisms.

In other cases, the phosphorylated residue appears to be conserved, but the surrounding sequence has diverged to the extent that a protein kinase with a different specificity would be required in the two organisms. One such example involves yeast Ace2p (phosphorylation site Ser701) and its human orthologue KLF14. The residues corresponding to the phosphorylated Ser91 and Ser96 in yeast Bud4p are Ser and Glu in the human orthologue claspin; if claspin Ser225 (the equivalent of Ser91 in Bud4p) was phosphorylated, it would require a protein kinase with a different specificity for phosphorylation, while the Glu provides a constitutive negative charge. Human enolase similarly has a glutamic acid in place of the yeast Eno1p phospho-Ser10.

There are cases where both the yeast and human orthologous proteins are known to be regulated by phosphorylation, but the mechanisms do not appear to be strictly conserved. Protein kinase Snf1 was predicted in this study to be responsible for phosphorylation of Acc1p (acetyl coenzyme A carboxylase) at Ser1157. Human acetyl coenzyme A carboxylase is known to be phosphorylated by AMPK at Ser1201 [64,65]. While the two serines are located in similar regions in their respective proteins, they do not strictly align [66]. Similarly, the regulatory subunit of PKA is a substrate for its catalytic subunit in both *S. cerevisiae *and mammalian proteins [15]. Again, the phosphorylated residue is in a similar location in the sequence, but they do not strictly align.

However, in many cases the region of the protein phosphorylated in yeast is poorly conserved in humans, or the equivalent region of the protein does not exist in the human protein. For example, in the human orthologue of the yeast protein Sec4p, the N-terminal phosphorylation sites are missing, while the C-terminal sites have diverged in sequence. The sequence surrounding the phosphorylation sites in yeast protein Abp1p is well conserved in the human orthologue mAbp1 [67]; however, the residues equivalent to the phosphorylated Thr181 and Ser183 have been substituted by amino acids other than Ser or Thr.

## Conclusion

In this study, we aimed to associate every phosphorylation site in the yeast phosphoproteome reported in the literature [4] with the protein kinase(s) most likely to be responsible for phosphorylation of that site. Our approach made use of the computer program Predikin, which is the only computational method that is able to shed light on the specificity of uncharacterized protein kinases [5,50]. The accuracy of Predikin-based predictions has been demonstrated previously using an experimental cross-validation set [5]. Moreover, several subsequent experimental confirmations of novel Predikin predictions have been reported [6-10]. As part of the present work, we also estimated the probabilities of individual predictions. We identified a possible kinase for most phosphorylation sites in the phosphoproteome dataset, more than half of the associations showing high probabilities. Certain classes of protein kinases have well-defined substrate specificities that make the associations more reliable; these include kinases in the AGC, CaMK, CMGC, MAPKK and CK1 groups. Because the phosphoproteome has been determined using yeast cells not subjected to any particular challenge, it is not surprising that certain groups of protein kinases such as the checkpoint kinases, were not predicted to be responsible for phosphorylation of any substrate in the dataset. On the other hand, housekeeping enzymes such as protein kinases CK1 and CK2 appear to have a number of substrates in the phosphoproteome dataset, supporting fundamental and constitutive roles in cell regulation for these kinases. Our analysis has created a foundation on which to base future experimental work, e.g. the effects of depletion or over-expression of the associated kinase on phosphorylation of the predicted substrate(s).

## Availability and requirements

Project name: Predikin; Project home page: ; Operating system: Platform-independent; Programming language: Java; Restrictions to use by academics: Registration needed; Restrictions to use by non-academics: Licence needed.

## Methods

### Association of the phosphoproteins with protein kinases

The procedure we used to associate each phosphorylation site with the protein kinase most likely to be responsible for that phosphorylation event consisted of the following steps.

1. The optimal heptapeptide sequences phosphorylated by the kinases were predicted using Predikin [5]. We considered a set of 116 protein kinases, based on the analysis of Hunter and Plowman [1], and additionally protein kinases Ylr253wp (Yl53p), Atg1p, and Yjl057cp. We did not include Scy1p into the analysis; although this protein is clearly related to protein kinases, it lacks the catalytic aspartate and some other conserved residues. The conserved sequence motifs were ambiguous in the case of Cak1p, Bub1p, Bud32p and Ygr262cp; this should be considered when interpreting the predictions for these kinases. Predictions cannot be carried out for phosphatidylinositol 3 kinase-like kinases (Tor1p and Mec1p) that are only distantly related to Ser/Thr protein kinases. Protein kinases Yck1p, Yck2p, Yck3p and Hrr25p also have identical predicted specificities and were designated as the "CK1 group". Protein kinases Cka1p, Cka2p and Cdc7p have the predicted specificities too similar to be distinguished, and were designated as the "CK2 group".

2. In the case of CMGC kinases (including CDK, MAPK, GSK3β and CLK families), the prediction rules strictly required a Pro residue at P+1 in the substrate. Similarly, the rules for CK2 family required that there was a Glu or Asp at P+1, and that there was a Glu, Ser, Ile or Gly (but not Asp) at P+1 for CK1.

3. The phosphopeptides were sorted and associated with the most likely protein kinase(s), using Scansite 1.5 scores [11] as a guide (the Quick Matrix option of Scansite 1.5 was used as described previously [5]. Scansite scores are not calculated accurately if the phosphorylation site is less than 7 residues from the terminus of the protein. Where no exact match between the phosphopeptide and the predicted motif was possible, the following process was used.

(i) Pro residue at (+1), together with Pro, Val, Leu, Ile, Thr or Ser at (-2) classified the kinase into CDK, MAPK, GSK3β or CLK families. A Pro residue at (+1) restricts the specificity to solely these protein kinases. The residues at other positions define the specific protein kinase. A large hydrophobic residue such as Phe at (-3) indicates the MAPKs Slt2p or Smk1p. Ser or other small hydrophilic residues at (-3), (+2) or (+3) indicate the MAPK Fus3p. Arg at (-3) indicates the class III_D kinase Yak1p. There is considerable overlap between the specificities of CDKs and MAPKs. Where there is no Pro at (+1), but Val or Gln instead, and the other residues fit the pattern for a CDK or MAPK, this indicates a GSK3β-like kinase such as Mck1p or Mds1p.

(ii) Arg residues at (-3) and (-2) classify the kinase into the AGC group, and the residues at (+1) and (+2) were used for more detailed assignment. Phe, Leu or Arg at (+1) (and other sites, particularly (+2)) indicate Pkc1p. If there is a Ser or Thr at (-3), this indicates Pkc1p only if there is Arg or Lys also present at (-2) and (+2). Smaller residues at (+1) and (+2) are indicative of a PKA-like kinase such as Tpk1p or Ypk1p. An acidic residue at (+2) indicates Ynr047wp, particularly when combined with Arg or Lys at (+1). Ser or Thr at (-3) may indicate an AGC kinase if there is Arg or Lys at (-2).

(iii) With Arg residue at (-3) only, the residue at (-2) (except for Ser, Thr or Leu) is used to define the protein kinase. Ser and Thr are compatible with either group I (AGC) or group II (CaMK) kinases and other sites must be examined. Ala, Val and Pro indicate either Snf1p or Cmk1p, and other residues such as Gln (but not Tyr, Glu or Trp), indicate Cmk1p. With these protein kinases, the (-3) residue is almost always Arg (Lys would make insufficient contacts). A large hydrophobic residue at (-2) and (+1) (e.g. Leu) indicates a protein kinase from family VI_D or VII (such as Chk1p or Mps1p).

(iv) With Glu predominating in sites (-3), (-2), (+1), (+2) and (+3), the kinase is from either the CK1 or the CK2 families (VI_A or VI_B). Ala and Gly at (+1) indicate CK1, while Asp at (+1) is only found with CK2. Phospho-serine or phospho-threonine residues in sites (-3), (-2), (+1), (+2) and (+3) are indicative of CK1. Phosphorylated residues are not acceptable in the (-1) site because of a clash with bound ATP, and this could often suggest the order of phosphorylation in multiply-phosphorylated peptides.

(v) Amino acids such as Gln, Ala, Gly, Leu or Tyr at (-3) indicate a kinase from families IV, V or VI_C-VI_F. A larger hydrophobic residue at (-3) is strongly indicative of family V_C (Ark1p or Prk1p), or possibly Gcn2p (family VII_A). These kinases are distinguished by the (+1) residue; Glu, Val, Thr or Tyr in the case of Ark1p and Prk1p, or Gly, Ser, Asp or Gly in the case of Gcn2p. Most of these kinases have a partially occluded (-2) pocket with a resulting specificity for Val, Ala, Thr or Pro. Specificity for Gln at (-2), on the other hand, indicates family IV_C (Cdc15p) or family V_A (Pbs2p), whereas (-2) specificity for small hydrophilic residues such as Asn or Ser signifies CDC15p (family IV_C). A smaller neutral or hydrophobic residue at (-2) suggests a family IV_A kinase, while a larger hydrophobic residue (such as Leu or Val) at (-2) suggests a family IV_C kinase (Sps1p) or a family VII_A kinase (Ybr097wp). The two alternatives can be distinguished by the (+2) residue specificity, which is for smaller residues in the case of family IV_C. An acidic residue at (+2) indicates Ste11p (family IV_A). A large hydrophobic residue, such as Phe, Leu, Ile or Met, at (+1) also indicates families IV_A (the Ste11p family), IV_B or VI_C. A hydrophilic residue (particularly a smaller one) indicates family V (the Ste20p family). A basic hydrophilic residue at (+1), such as Arg, indicates family VI_C (the Hal5p family). In all cases, residues with larger side chains represent more definitive specificity than those with smaller side chains.

(vi) For the few phosphopeptides that did not satisfy any of the criteria described above, the sequences were sorted according to the amino acids in positions (-3), (-2), (+1), (+2) and (+3) (in the order of decreasing constraints in subsite specificity), with the amino acids grouped as basic, acidic, neutral hydrophilic, small hydrophilic, small hydrophobic, and large hydrophobic (Val or larger), and compared with the predicted kinase specificities. The amino acid preferences for a particular subsite in a kinase can usually be grouped into primary (utilizing the majority of available interactions), secondary (making favourable interactions but not taking advantage of the optimal number of contacts), and compatible (making few or no interactions with the kinase, but compatible with the polarity of the environment; usually amino acids such as Asn, Ser, Pro, Ala and Gly). Small hydrophobic and small hydrophilic residues can usually be accommodated equally well. Accurate predictions are not possible when the phosphoresidue is less than 4 residues from the N- or C-terminus of a protein.

4. The probabilities of predictions were estimated based on the Scansite 1.5 scores (based on the relationships derived as described in the next section). Characterized phosphorylation sites can exhibit a poor match with the optimal phosphorylation sequence of a kinase [5,11]; therefore, it is possible that some phosphorylation sites are substrates for other kinase(s) with similar but weaker match to the optimal specificities.

5. Information on the functions of the phosphoproteins and protein kinases was obtained from various databases (RefSeq [68], Swissprot [69] and others) and literature searches.

### Estimation of prediction probabilities

To estimate the probabilities of the predictions of phosphorylation sites in known substrates, we analysed the Scansite scores for all possible phosphorylation sites in a substrate, using a dataset of known protein kinase – substrate pairs (Phosphobase, [70]). We compared the Scansite scores of sites that are phosphorylated to the Scansite scores of sites that are not phosphorylated (Table 6). The serine and threonine residues that are known not to be phosphorylated yielded a mean Scansite score of 0.260 ± 0.050, while the sites known to be phosphorylated yielded a mean Scansite score of 0.135 ± 0.045. Only 4 out of 558 sites with scores below 0.128 were not phosphorylated, and 10 out of 52 known phosphorylation sites had scores above 0.212 (Figure 1).

**Table 6:** Protein kinase- substrate pairs used in the estimating the probabilities of predictions based on Scansite scores

Protein kinase *	Substrate protein (source)	Number of all serine and threonine residues	Number of known phosphorylation sites
PKA	Myelin basic protein (bovine)	22	6
PKA	Vimentin (mouse)	64	5
PKA	Vasodilator-stimulated phosphoprotein (human)	41	3
PKC	Pleckstrin (human)	36	3
PKC	MARCKS (human)	35	3
CaMK2	Myelin basic protein (bovine)	22	4
CaMK2	Vimentin (mouse)	64	2
CK2	DARPP-32 (bovine)	22	3
CK2	Alpha-S1 casein (bovine)	19	3
CK2	DNA topoisomerase II (S. cerevisiae)	172	9
CK1	Alpha-S2 casein (bovine)	30	6
CK1	Glycogen synthase, muscle (rabbit)	93	4

* Protein kinase-substrate pairs selected from Phosphobase [70].

To estimate the probability of a particular protein kinase being associated with a particular phosphorylation site, we require a different analysis that involves the examination of the range of scores obtained for known substrates using a diverse set of kinases. We analysed all the substrates listed in Phosphobase [70] for 8 diverse protein kinases (PKA, PKC, CaMK2, PHK, CDK1, MAPK, CK1 and CK2). There is n% probability that a kinase is responsible for a phosphorylation at a site yielding a particular Scansite score, when (100-n)% phosphorylation sites have Scansite scores lower or equal to that particular score (Figure 1).

## Authors' contributions

RIB carried out most of the data acquisition and analysis and participated in the design of the study, and preparation of the manuscript. ALM participated in data analysis and interpretation, and preparation of the manuscript. BK conceived the study, participated in data acquisition, analysis and interpretation, and preparation of the manuscript. All authors read and approved the final manuscript.

## Supplementary Material

Additional File 1 Table S1: Substrate specificities of *S. cerevisiae *protein kinases Click here for file

Additional File 2 Table S2: The *S. cerevisiae *phosphoproteome Click here for file

Additional File 3 Table S3: Protein kinases associated with *S. cerevisiae *phosphoproteins Click here for file

Additional File 4 Table S4: Selected protein kinase- phosphoprotein pairs suggesting functional roles for uncharacterized proteins Click here for file

## References

[B1] Hunter T, Plowman GD (1997). The protein kinases of budding yeast: six score and more. Trends Biochem Sci.

[B2] Stern DF, Zheng P, Beidler DR, Zerillo C (1991). Spk1, a new kinase from Saccharomyces cerevisiae, phosphorylates proteins on serine, threonine, and tyrosine. Mol Cell Biol.

[B3] Zhu H, Klemic JF, Chang S, Bertone P, Casamayor A, Klemic KG, Smith D, Gerstein M, Reed MA, Snyder M (2000). Analysis of yeast protein kinases using protein chips. Nature Genet.

[B4] Ficarro SB, McCleland ML, Stukenberg PT, Burke DJ, Ross MM, Shabanowitz J, Hunt DF, White FM (2002). Phosphoproteome analysis by mass spectrometry and its application to Saccharomyces cerevisiae. Nature Biotechnol.

[B5] Brinkworth RI, Breinl RA, Kobe B (2003). Structural basis and prediction of substrate specificity in protein serine/threonine kinases. Proc Natl Acad Sci USA.

[B6] Dinudom A, Fotia AB, Lefkowitz RJ, Young JA, Kumar S, Cook DI (2004). The kinase Grk2 regulates Nedd4/Nedd4-2-dependent control of epithelial Na+ channels. Proc Natl Acad Sci USA.

[B7] Court NW, Kuo I, Quigley O, Bogoyevitch MA (2004). Phosphorylation of the mitochondrial protein Sab by stress-activated protein kinase 3. Biochem Biophys Res Commun.

[B8] Fujii K, Zhu G, Liu Y, Hallam J, Chen L, Herrero J, Shaw S (2004). Kinase peptide specificity: improved determination and relevance to protein phosphorylation. Proc Natl Acad Sci USA.

[B9] Poon IK, Oro C, Dias MM, Zhang J, Jans DA (2005). Apoptin nuclear accumulation is modulated by a CRM1-recognized nuclear export signal that is active in normal but not in tumor cells. Cancer Res.

[B10] Kelner A, Pekala I, Kaczanowski S, Muszynska G, Hardie DG, Dobrowolska G (2004). Biochemical characterization of the tobacco 42-kD protein kinase activated by osmotic stress. Plant Physiol.

[B11] Yaffe MB, Leparc GG, Lai J, Obata T, Volinia S, Cantley LC (2001). A motif-based profile scanning approach for genome-wide prediction of signaling pathways. Nature Biotechnol.

[B12] Friesen H, Murphy K, Breitkreutz A, Tyers M, Andrews B (2003). Regulation of the yeast amphiphysin homologue Rvs167p by phosphorylation. Mol Biol Cell.

[B13] Garrett S, Broach J (1989). Loss of Ras activity in Saccharomyces cerevisiae is suppressed by disruptions of a new kinase gene, YAKI, whose product may act downstream of the cAMP-dependent protein kinase. Genes Dev.

[B14] Erlichman J, Rosenfeld R, Rosen OM (1974). Phosphorylation of a cyclic adenosine 3':5'-monophosphate-dependent protein kinase from bovine cardiac muscle. J Biol Chem.

[B15] Diella F, Cameron S, Gemund C, Linding R, Via A, Kuster B, Sicheritz-Ponten T, Blom N, Gibson TJ (2004). Phospho.ELM: a database of experimentally verified phosphorylation sites in eukaryotic proteins. BMC Bioinformatics.

[B16] Mitchelhill KI, Stapleton D, Gao G, House C, Michell BJ, Katsis F, Witters LA, Kemp BE (1994). Mammalian AMP-activated protein kinase shares structural and functional homology with the catalytic domain of yeast snf1 protein kinase. J Biol Chem.

[B17] Huang D, Wilson WA, Roach PJ (1997). Glucose-6-P control of glycogen synthase phosphorylation in yeast. J Biol Chem.

[B18] Bardwell L, Cook JG, Chang EC, Cairns BR, Thorner J (1996). Signaling in the yeast pheromone response pathway: specific and high-affinity interaction of the mitogen-activated protein (MAP) kinases Kss1 and Fus3 with the upstream MAP kinase kinase Ste7. Mol Cell Biol.

[B19] Tatebayashi K, Takekawa M, Saito H (2003). A docking site determining specificity of Pbs2 MAPKK for Ssk2/Ssk22 MAPKKKs in the yeast HOG pathway. EMBO J.

[B20] Mortensen EM, McDonald H, Yates J, Kellogg DR (2002). Cell cycle-dependent assembly of a Gin4-septin complex. Mol Biol Cell.

[B21] Wojda I, Alonso-Monge R, Bebelman JP, Mager WH, Siderius M (2003). Response to high osmotic conditions and elevated temperature in Saccharomyces cerevisiae is controlled by intracellular glycerol and involves coordinate activity of MAP kinase pathways. Microbiol.

[B22] Conlan RS, Tzamarias D (2001). Sfl1 functions via the co-repressor Ssn6-Tup1 and the cAMP-dependent protein kinase Tpk2. J Mol Biol.

[B23] Lagorce A, Hauser NC, Labourdette D, Rodriguez C, Martin-Yken H, Arroyo J, Hoheisel JD, Francois J (2003). Genome-wide analysis of the response to cell wall mutations in the yeast Saccharomyces cerevisiae. J Biol Chem.

[B24] Ward MP, Garrett S (1994). Suppression of a yeast cyclic AMP-dependent protein kinase defect by overexpression of SOK1, a yeast gene exhibiting sequence similarity to a developmentally regulated mouse gene. Mol Cell Biol.

[B25] Rempola B, Kaniak A, Migdalski A, Rytka J, Slonimski PP, di Rago JP (2000). Functional analysis of RRD1 (YIL153w) and RRD2 (YPL152w), which encode two putative activators of the phosphotyrosyl phosphatase activity of PP2A in Saccharomyces cerevisiae. Mol Gen Genet.

[B26] Giot L, DeMattei C, Konopka JB (1999). Combining mutations in the incoming and outgoing pheromone signal pathways causes a synergistic mating defect in Saccharomyces cerevisiae. Yeast.

[B27] Hicke L, Zanolari B, Riezman H (1998). Cytoplasmic tail phosphorylation of the alpha-factor receptor is required for its ubiquitination and internalization. J Cell Biol.

[B28] Dunn R, Hicke L (2001). Multiple roles for Rsp5p-dependent ubiquitination at the internalization step of endocytosis. J Biol Chem.

[B29] Stamenova SD, Dunn R, Adler AS, Hicke L (2004). The Rsp5 ubiquitin ligase binds to and ubiquitinates members of the yeast CIN85-endophilin complex, Sla1-Rvs167. J Biol Chem.

[B30] Dewar H, Warren DT, Gardiner FC, Gourlay CG, Satish N, Richardson MR, Andrews PD, Ayscough KR (2002). Novel proteins linking the actin cytoskeleton to the endocytic machinery in Saccharomyces cerevisiae. Mol Biol Cell.

[B31] Tang HY, Xu J, Cai M (2000). Pan1p, End3p, and S1a1p, three yeast proteins required for normal cortical actin cytoskeleton organization, associate with each other and play essential roles in cell wall morphogenesis. Mol Cell Biol.

[B32] Gagny B, Wiederkehr A, Dumoulin P, Winsor B, Riezman H, Haguenauer-Tsapis R (2000). A novel EH domain protein of Saccharomyces cerevisiae, Ede1p, involved in endocytosis. J Cell Sci.

[B33] van Delft S, Govers R, Strous GJ, Verkleij AJ, van Bergen en Henegouwen PM (1997). Epidermal growth factor induces ubiquitination of Eps15. J Biol Chem.

[B34] Polo S, Sigismund S, Faretta M, Guidi M, Capua MR, Bossi G, Chen H, De Camilli P, Di Fiore PP (2002). A single motif responsible for ubiquitin recognition and monoubiquitination in endocytic proteins. Nature.

[B35] Guo W, Roth D, Walch-Solimena C, Novick P (1999). The exocyst is an effector for Sec4p, targeting secretory vesicles to sites of exocytosis. EMBO J.

[B36] Price A, Seals D, Wickner W, Ungermann C (2000). The docking stage of yeast vacuole fusion requires the transfer of proteins from a cis-SNARE complex to a Rab/Ypt protein. J Cell Biol.

[B37] LaGrassa TJ, Ungermann C (2005). The vacuolar kinase Yck3 maintains organelle fragmentation by regulating the HOPS tethering complex. J Cell Biol.

[B38] Novick P, Botstein D (1985). Phenotypic analysis of temperature-sensitive yeast actin mutants. Cell.

[B39] Mulholland J, Wesp A, Riezman H, Botstein D (1997). Yeast actin cytoskeleton mutants accumulate a new class of Golgi-derived secretary vesicle. Mol Biol Cell.

[B40] Breton AM, Schaeffer J, Aigle M (2001). The yeast Rvs161 and Rvs167 proteins are involved in secretory vesicles targeting the plasma membrane and in cell integrity. Yeast.

[B41] Winsor B, Schiebel E (1997). Review: an overview of the Saccharomyces cerevisiae microtubule and microfilament cytoskeleton. Yeast.

[B42] Amberg DC (1998). Three-dimensional imaging of the yeast actin cytoskeleton through the budding cell cycle. Mol Biol Cell.

[B43] Lew DJ, Reed SI (1993). Morphogenesis in the yeast cell cycle: regulation by Cdc28 and cyclins. J Cell Biol.

[B44] Drubin DG, Miller KG, Botstein D (1988). Yeast actin-binding proteins: evidence for a role in morphogenesis. J Cell Biol.

[B45] Humphries CL, Balcer HI, D'Agostino JL, Winsor B, Drubin DG, Barnes G, Andrews BJ, Goode BL (2002). Direct regulation of Arp2/3 complex activity and function by the actin binding protein coronin. J Cell Biol.

[B46] Goode BL, Rodal AA, Barnes G, Drubin DG (2001). Activation of the Arp2/3 complex by the actin filament binding protein Abp1p. J Cell Biol.

[B47] Schmidt A, Kunz J, Hall MN (1996). TOR2 is required for organization of the actin cytoskeleton in yeast. Proc Natl Acad Sci USA.

[B48] Beck T, Schmidt A, Hall MN (1999). Starvation induces vacuolar targeting and degradation of the tryptophan permease in yeast. J Cell Biol.

[B49] Uetz P, Giot L, Cagney G, Mansfield TA, Judson RS, Knight JR, Lockshon D, Narayan V, Srinivasan M, Pochart P, Qureshi-Emili A, Li Y, Godwin B, Conover D, Kalbfleisch T, Vijayadamodar G, Yang M, Johnston M, Fields S, Rothberg JM (2000). A comprehensive analysis of protein-protein interactions in Saccharomyces cerevisiae. Nature.

[B50] Kobe B, Kampmann T, Forwood JK, Listwan P, Brinkworth RI (2005). Substrate specificity of protein kinases and computational prediction of substrates. Biochim Biophys Acta.

[B51] Malathi K, Xiao Y, Mitchell AP (1999). Catalytic roles of yeast GSK3beta/shaggy homolog Rim11p in meiotic activation. Genetics.

[B52] Dhanasekaran N, Premkumar Reddy E (1998). Signaling by dual specificity kinases. Oncogene.

[B53] Kiegerl S, Cardinale F, Siligan C, Gross A, Baudouin E, Liwosz A, Eklof S, Till S, Bogre L, Hirt H, Meskiene I (2000). SIMKK, a mitogen-activated protein kinase (MAPK) kinase, is a specific activator of the salt stress-induced MAPK, SIMK. Plant Cell.

[B54] Fleming Y, Armstrong CG, Morrice N, Paterson A, Goedert M, Cohen P (2000). Synergistic activation of stress-activated protein kinase 1/c-Jun N-terminal kinase (SAPK1/JNK) isoforms by mitogen-activated protein kinase kinase 4 (MKK4) and MKK7. Biochem J.

[B55] Mendenhall MD, Hodge AE (1998). Regulation of Cdc28 cyclin-dependent protein kinase activity during the cell cycle of the yeast Saccharomyces cerevisiae. Microbiol Mol Biol Rev.

[B56] John PC, Mews M, Moore R (2001). Cyclin/Cdk complexes: their involvement in cell cycle progression and mitotic division. Protoplasma.

[B57] Alfarano C, Andrade CE, Anthony K, Bahroos N, Bajec M, Bantoft K, Betel D, Bobechko B, Boutilier K, Burgess E, Buzadzija K, Cavero R, D'Abreo C, Donaldson I, Dorairajoo D, Dumontier MJ, Dumontier MR, Earles V, Farrall R, Feldman H, Garderman E, Gong Y, Gonzaga R, Grytsan V, Gryz E, Gu V, Haldorsen E, Halupa A, Haw R, Hrvojic A, Hurrell L, Isserlin R, Jack F, Juma F, Khan A, Kon T, Konopinsky S, Le V, Lee E, Ling S, Magidin M, Moniakis J, Montojo J, Moore S, Muskat B, Ng I, Paraiso JP, Parker B, Pintilie G, Pirone R, Salama JJ, Sgro S, Shan T, Shu Y, Siew J, Skinner D, Snyder K, Stasiuk R, Strumpf D, Tuekam B, Tao S, Wang Z, White M, Willis R, Wolting C, Wong S, Wrong A, Xin C, Yao R, Yates B, Zhang S, Zheng K, Pawson T, Ouellette BF, Hogue CW (2005). The Biomolecular Interaction Network Database and related tools 2005 update. Nucleic Acids Res.

[B58] Ho Y, Gruhler A, Heilbut A, Bader GD, Moore L, Adams SL, Millar A, Taylor P, Bennett K, Boutilier K, Yang L, Wolting C, Donaldson I, Schandorff S, Shewnarane J, Vo M, Taggart J, Goudreault M, Muskat B, Alfarano C, Dewar D, Lin Z, Michalickova K, Willems AR, Sassi H, Nielsen PA, Rasmussen KJ, Andersen JR, Johansen LE, Hansen LH, Jespersen H, Podtelejnikov A, Nielsen E, Crawford J, Poulsen V, Sorensen BD, Matthiesen J, Hendrickson RC, Gleeson F, Pawson T, Moran MF, Durocher D, Mann M, Hogue CW, Figeys D, Tyers M (2002). Systematic identification of protein complexes in Saccharomyces cerevisiae by mass spectrometry. Nature.

[B59] Gavin AC, Bosche M, Krause R, Grandi P, Marzioch M, Bauer A, Schultz J, Rick JM, Michon AM, Cruciat CM, Remor M, Hofert C, Schelder M, Brajenovic M, Ruffner H, Merino A, Klein K, Hudak M, Dickson D, Rudi T, Gnau V, Bauch A, Bastuck S, Huhse B, Leutwein C, Heurtier MA, Copley RR, Edelmann A, Querfurth E, Rybin V, Drewes G, Raida M, Bouwmeester T, Bork P, Seraphin B, Kuster B, Neubauer G, Superti-Furga G (2002). Functional organization of the yeast proteome by systematic analysis of protein complexes. Nature.

[B60] Ito T, Chiba T, Ozawa R, Yoshida M, Hattori M, Sakaki Y (2001). A comprehensive two-hybrid analysis to explore the yeast protein interactome. Proc Natl Acad Sci USA.

[B61] von Mering C, Krause R, Snel B, Cornell M, Oliver SG, Fields S, Bork P (2002). Comparative assessment of large-scale data sets of protein-protein interactions. Nature.

[B62] Ptacek J, Devgan G, Michaud G, Zhu H, Zhu X, Fasolo J, Guo H, Jona G, Breitkreutz A, Sopko R, McCartney RR, Schmidt MC, Rachidi N, Lee SJ, Mah AS, Meng L, Stark MJ, Stern DF, De Virgilio C, Tyers M, Andrews B, Gerstein M, Schweitzer B, Predki PF, Snyder M (2005). Global analysis of protein phosphorylation in yeast. Nature.

[B63] Bell M, Capone R, Pashtan I, Levitzki A, Engelberg D (2001). Isolation of hyperactive mutants of the MAPK p38/Hog1 that are independent of MAPK kinase activation. J Biol Chem.

[B64] Munday MR, Campbell DG, Carling D, Hardie DG (1988). Identification by amino acid sequencing of three major regulatory phosphorylation sites on rat acetyl-CoA carboxylase. Eur J Biochem.

[B65] Mohamed AH, Huang WY, Huang W, Venkatachalam KV, Wakil SJ (1994). Isolation and characterization of a novel acetyl-CoA carboxylase kinase from rat liver. J Biol Chem.

[B66] Chenna R, Sugawara H, Koike T, Lopez R, Gibson TJ, Higgins DG, Thompson JD (2003). Multiple sequence alignment with the Clustal series of programs. Nucleic Acids Res.

[B67] Kessels MM, Engqvist-Goldstein AE, Drubin DG, Qualmann B (2001). Mammalian Abp1, a signal-responsive F-actin-binding protein, links the actin cytoskeleton to endocytosis via the GTPase dynamin. J Cell Biol.

[B68] Pruitt KD, Tatusova T, Maglott DR (2003). NCBI Reference Sequence project: update and current status. Nucleic Acids Res.

[B69] Boeckmann B, Bairoch A, Apweiler R, Blatter MC, Estreicher A, Gasteiger E, Martin MJ, Michoud K, O'Donovan C, Phan I, Pilbout S, Schneider M (2003). The SWISS-PROT protein knowledgebase and its supplement TrEMBL in 2003. Nucleic Acids Res.

[B70] Kreegipuu A, Blom N, Brunak S (1999). PhosphoBase, a database of phosphorylation sites: release 2.0. Nucleic Acids Res.

