# Supplementary material for: Protein kinases associated with the yeast phosphoproteome
Source: BMC Bioinformatics. 2006 Jan 31;7:47. doi: 10.1186/1471-2105-7-47 (PMC1373605; doi:10.1186/1471-2105-7-47)
Supplement: Additional File 4 — Table S4: Selected protein kinase- phosphoprotein pairs suggesting functional roles for uncharacterized proteins [file 1471-2105-7-47-S4.doc]

## Table S4. Selected protein kinase- phosphoprotein pairs suggesting functional roles for uncharacterized proteins

| Substrate  protein | Function * | Predicted  protein  kinase | Function * | Phosphopeptide  ** | Scansite  score  *** | Estimated  probability  **** |
| --- | --- | --- | --- | --- | --- | --- |
| Ncb2p | Transcriptional regulation | Akl1p | Protein kinase (unknown function) | HHNSVSD | 0.256 | 0.1 |
| Akl1p | Protein kinase (unknown function) | CK1 group ***** | Cellular morphogenesis, cytokinesis, endocytosis, DNA repair, cell growth, chromosome segregation, meiosis, mitosis, nuclear division | DKDSNSSITI | 0.124, 0.144 | 0.75, 0.6 |
| Pma1p, Pma2p | Proton pump, regulation of pH | Kin4p | Unknown | RVSTQHE | — ******* |  |
| Ste2p | Pheromone alpha factor receptor | Kns1p | Unknown | DMY**T**PDTAAD | 0.207 | 0.2 |
| Cho1p | Phosphatidylserine synthase | Ksp1p | Protein kinase (unknown function) | GTLSRRA | 0.143 | 0.6 |
| Boi2p | Phospholipid binding, cell polarity | Ksp1p | Protein kinase (unknown function) | PSPTRNS | 0.121 | 0.75 |
| Tsl1p | Trehalose phosphate synthase (carbohydrate metabolism, stress response) | Ksp1p | Protein kinase (unknown function) | RSA**T**RSPSA | 0.146 | 0.6 |
| Ypr156cp | Polyamine transport | Ksp1p | Protein kinase (unknown function) | RTSTAI**S**RTR | 0.144 | 0.6 |
| Ksp1p | Protein kinase (unknown function) | CK1 group | Cellular morphogenesis, cytokinesis, endocytosis, DNA repair, cell growth, chromosome segregation, meiosis, mitosis, nuclear division | NESSSTSPDE | 0.144, 0.062 | 0.6, 0.9 |
| Ydr466wp | Protein kinase (unknown function) | Mek1p | Meiosis | SKASSEP | 0.082 | 0.85 |
| Ydr466wp | Protein kinase (unknown function) | Cdc28p | Cyclin-dependent protein kinase, regulation of mitosis and meiosis | EPSSPPP | 0.02 | 0.99 |
| Pda1p | Pyruvate dehydrogenase component (pyruvate metabolism) | Ygl179cp | Unknown | GGHSMSD | 0.143 | 0.6 |
| Sgv1p | Protein kinase (transcription) | Yjl057wp | Unknown | AKYTSVV | 0.255 | 0.1 |
| Chs2p | Chitin synthase (cytokinesis, osmotic stress response) | Ypr106wp | Unknown | YRDSAHN | 0.143 | 0.6 |
| Sol2p | Phospholuconolactonase (tRNA processing) | Ypr106wp | Unknown | CKS**T**ASAAE | 0.187 | 0.33 |
| Bre5p | Unknown | Cdc28p | Cyclin-dependent protein kinase, regulation of mitosis and meiosis | NAS**T**PSSSPE | 0.165 | 0.5 |
| Bre5p | Unknown | Kcc4p | Axial budding, septin ring assembly | NASTPS**S**SPE | 0.1 | 0.75 |
| Mlf3p | Unknown | Ume5p | Cyclin-dependent protein kinase, meiosis, regulation of transcription (Pol II promoter) | PATSPYV | 0.084 | 0.85 |
| Mlf3p | Unknown | Ume5p | Cyclin-dependent protein kinase, meiosis, regulation of transcription (Pol II promoter) | PYVSPQQ | 0.099 | 0.8 |
| Mrh1p | Unknown | Ctk1p | Protein kinase (transcriptional regulation (Pol II promoter)) | PAATPNL | 0.145 | 0.6 |
| Mrh1p | Unknown | Cdc28p | Cyclin-dependent protein kinase, regulation of mitosis and meiosis | PVASPRP | 0.063 | 0.9 |
| Ycr023cp | Unknown | Pkc1p | Actin filament organization, cell wall organization and biogenesis | HRS**S**LSSLSN | 0.101 | 0.8 |
| Ycr023cp | Unknown | CK1 group | Cellular morphogenesis, cytokinesis, endocytosis, DNA repair, cell growth, chromosome segregation, meiosis, mitosis, nuclear division | HRSSLS**S**LSN | 0.125 | 0.75 |
| Ydl189wp | Unknown | Mek1p | Meiosis | RRASVEG | 0.064 | 0.9 |
| Ydl189wp | Unknown | Ume5p | Cyclin-dependent protein kinase, meiosis, regulation of transcription (Pol II promoter) | VEGSPSS | 0.1 | 0.8 |
| Ydr090cp | Unknown | Pkc1p | Actin filament organization, cell wall organization and biogenesis | SRLSV-- | — ******* |  |
| Yfr016cp | Unknown | Ume5p | Cyclin-dependent protein kinase, meiosis, regulation of transcription (Pol II promoter) | TPESPKV | 0.02 | 0.99 |
| Yfr017cp | Unknown | Mek1p | Meiosis | RRRSTNY | 0.127 | 0.75 |
| Yfr017cp | Unknown | Tpk1p | cAMP-dependent protein kinase, Ras signal transduction, pseudohyphal growth | RRSSGPM | 0.19 | 0.33 |
| Yfr024cp | Unknown | Gcn2p | Amino acid biosynthesis, regulation of translational initiation | PTN**S**GGSGGK | 0.233 | 0.05 |
| Yfr024cp | Unknown | Gcn2p | Amino acid biosynthesis, regulation of translational initiation | PTNSGG**S**GGK | 0.143 | 0.6 |
| Yhr097cp | Unknown | Rck2p | Regulation of meiosis | ANS**S**TTTLD | 0.077 | 0.85 |
| Yhr097cp | Unknown | CK1 group | Cellular morphogenesis, cytokinesis, endocytosis, DNA repair, cell growth, chromosome segregation, meiosis, mitosis, nuclear division | ANSST**T**TLD | 0.206 | 0.33 |
| Yhr132wp | Unknown | Tpk1p | cAMP-dependent protein kinase, Ras signal transduction, pseudohyphal growth | RRM**S**SSSG | 0.101 | 0.75 |
| Yhr132wp | Unknown | Cmk2p | Calmodulin-dependent protein kinase | RRMS**S**SSG | 0.1 | 0.8 |
| *Yhr186cp | Unknown | Pbs2p | Actin filament organization, osmoregulation | KAGSIQ**T**QSR | 0.104 | 0.8 |
| Yhr186cp | Unknown | Prk1p | Actin filament organization, cytokinesis | ANL**S**TMSLVN | 0.167 | 0.5 |
| Yhr186cp | Unknown | Ssk2p | MAPKKK, actin cytoskeleton organization and biogenesis, osmosensory signaling pathway | ANLSTM**S**LVN | 0.038 | 0.95 |
| Yml029wp | Unknown | Yak1p | Cell growth and maintenance | RSQ**S**PVSFAP | 0.084 | 0.85 |
| Yml029wp | Unknown | Sps1p | Cell wall organization and biogenesis, cellular morphogenesis | RSQSPV**S**FAP | 0.145 | 0.6 |
| Yml072cp | Unknown | Cdc28p | Cyclin-dependent protein kinase, regulation of mitosis and meiosis | TSVTPRA | 0.101 | 0.8 |
| Yml072cp | Unknown | Mps1p | Mitotic spindle checkpoint, spindle pole body duplication | RASSFAR | 0.128 | 0.75 |
| Ymr196wp | Unknown | Mck1p | Double-strand break repair, meiosis, mitotic chromosome segregation, response to stress, sporulation | SGLTPQS | 0.102 | 0.8 |
| Ymr196wp | Unknown | Gcn2p | Amino acid biosynthesis, regulation of translational initiation | SISSDKA | 0.124 | 0.75 |
| Ymr295cp | Unknown | CK1 group | Cellular morphogenesis, cytokinesis, endocytosis, DNA repair, cell growth, chromosome segregation, meiosis, mitosis, nuclear division | SSI**S**NTSDHD | 0.082 | 0.85 |
| Ymr295cp | Unknown | CK2 group ****** | Regulation of DNA replication, G1/S and G2/M transition of mitotic cell cycle, cell ion homeostasis, cell polarity, flocculation, regulation of transcription Pol I and Pol III promoters), response to DNA damage | SSISNT**S**DHD | 0.168 | 0.5 |
| Ynl136wp | Unknown | Ark1p | Actin filament organization, cytokinesis | GNTSNET | 0.122 | 0.75 |
| Ynl136wp | Unknown | Mck1p | Double-strand break repair, meiosis, mitotic chromosome segregation, response to stress, sporulation | NETSPKR | 0.081 | 0.85 |
| Ynl156cp | Unknown | Cmk2p | Calmodulin-dependent protein kinase | RSV**S**IDSTKY | 0.099 | 0.8 |
| Ynl156cp | Unknown | Cdc15p | Cytokinesis, regulation of exit from mitosis | RSVSID**S**TKY | 0.146 | 0.6 |
| Ynl156cp | Unknown | Cmk2p | Calmodulin-dependent protein kinase | RIL**S**ASSIHE | 0.119 | 0.75 |
| Ynl321wp | Unknown | Cdc15p | Cytokinesis, regulation of exit from mitosis | ATPSSPK | 0.165 | 0.5 |
| Yor042wp | Unknown | Ste11p | MAPKKK, pseudohyphal growth, conjugation with cellular fusion | YID**T**PDTETK | 0.058 | 0.9 |
| Yor042wp | Unknown | Bub1p | Mitotic spindle checkpoint | YIDTPD**T**ETK | 0.149 | 0.6 |
| Yor052cp | Unknown | Ykl116cp | Receptor signalling, MAPK cascade | RSS**S**NSSVTS | 0.039 | 0.95 |
| Yor052cp | Unknown | CK1 group | Cellular morphogenesis, cytokinesis, endocytosis, DNA repair, cell growth, chromosome segregation, meiosis, mitosis, nuclear division | RSSSNS**S**VTS | 0.082 | 0.85 |
| Yor175cp | Unknown | Gcn2p | Amino acid biosynthesis, regulation of translational initiation | MSF**S**GYSPKP | 0.233 | 0.05 |
| Yor175cp | Unknown | Mck1p | Double-strand break repair, meiosis, mitotic chromosome segregation, response to stress, sporulation | MSFSGY**S**PKP | 0.082 | 0.85 |
| Ypl247cp | Unknown | Ypk1p | Endocytosis, sphingolipid metabolism | KRSSISF | 0.083 | 0.85 |
| Yro2p | Unknown (heat shock protein homologue) | CK2 group | Regulation of DNA replication, G1/S and G2/M transition of mitotic cell cycle, cell ion homeostasis, cell polarity, flocculation, regulation of transcription Pol I and Pol III promoters), response to DNA damage | DVA**T**D**S**E-- | — ^*******^ |  |
| Sec31p | Endoplasmic reticulum-Golgi transport, autophagy | Atg1p | Vesicle organization and biogenesis, autophagy | RVPSLVA | 0.062 | 0.9 |

* Functional annotations based on RefSeq (Pruitt et al. 2003)

** The phosphorylated residues are underlined, and the residues not present in the phosphopeptide sequences (Ficarro et al. 2002) are shown in italic. When there is more than one phosphorylation site, the one discussed is shown in bold, unless the same protein kinase is predicted for all sites in the peptide.

*** Scansite (Yaffe et al. 1999) scores were calculated as described in the Methods section. When the same protein kinase is predicted for more than one site in the peptide, the scores are given for the respective sites, starting at the N-terminus. If more than one protein kinase yields a similar score, all the possible kinases are listed.

**** Probabilities were calculated as described in the Methods section (Figure 1). When the same protein kinase is predicted for more than one site in the peptide, the values are given for the respective sites, starting at the N-terminus.

***** Protein kinases Cka1p, Cka2p or Cdc7p. The predicted specificities are too similar to be distinguished.

****** Protein kinases Yck1p, Yck2p, Yck3p or Hrr25p. The predicted specificities are identical.

******* Scansite score could not be measured because the phosphorylation site is too close to the C-terminus.
